# Exploring the Chemopreventive Effect of Medication on Gene Expression Linked to Colorectal Cancer: An Observational and Mendelian Randomization Analysis in Healthy Colon Mucosa

**DOI:** 10.3390/ijms252111395

**Published:** 2024-10-23

**Authors:** Ferran Moratalla-Navarro, Robert Carreras-Torres, Virginia Díez-Obrero, Matthew Devall, Mireia Obón-Santacana, Anna Díez-Villanueva, Elisabet Guinó, Graham Casey, Li Li, Victor Moreno

**Affiliations:** 1 Oncology Data Analytics Program, Catalan Institute of Oncology, 08908 L’Hospitalet de Llobregat, Barcelona, Spain; 2 Colorectal Cancer Group, Molecular Mechanisms and Experimental Therapy in Oncology (ONCOBELL) Program, Bellvitge Biomedical Research Institute, 08908 L’Hospitalet de Llobregat, Barcelona, Spain; rcarreras@idibgi.org; 3 Consortium for Biomedical Research in Epidemiology and Public Health, 28029 Madrid, Spain; 4 Department of Clinical Sciences, Faculty of Medicine and Health Sciences and Universitat de Barcelona Institute of Complex Systems (UBICS), University of Barcelona (UB), 08907 L’Hospitalet de Llobregat, Barcelona, Spain; 5 Group of Digestive Diseases and Microbiota, Institute d’Investigació Biomèdica de Girona Dr Josep Trueta-IDIBGI, 17190 Salt, Girona, Spain; 6 Department of Family Medicine, University of Virginia, Charlottesville, VA 22903, USA; 7 Department of Genome Sciences, University of Virginia, Charlottesville, VA 22903, USA

**Keywords:** colorectal cancer, tumor microenvironment, chemoprevention, gene expression, Mendelian randomization

## Abstract

Gene expression appears altered in apparently normal tissue surrounding tumor tissue. The observed biological alterations in the tumor microenvironment play a crucial role in cancer development and are named the cancer field effect (FE). A robust set of overexpressed FE genes in tissue surrounding colorectal cancer (CRC) tumor were identified in previous studies. Our study aimed to investigate the influence of common medication intake and modifiable risk factors on FE gene expression using a colonic mucosa sample dataset of healthy individuals (BarcUVa-Seq). We applied expression enrichment analysis of the FE genes for each studied medication and factor. Both observational and instrumental (Mendelian randomization) analysis were conducted, and the results were validated using independent datasets. The findings from the observational and instrumental analyses consistently showed that medication intake, especially metformin, considerably downregulated the FE genes. Chemopreventive effects were also noted for antihypertensive drugs targeting the renin–angiotensin system. Conversely, benzodiazepines usage might upregulate FE genes, thus fostering a tumor-promoting microenvironment. In contrast, the findings from the observational and instrumental analyses on modifiable risk factors showed some discrepancies. The instrumental results indicated that obesity and smoking might promote a tumor-favorable microenvironment. These findings offer insights into the biological mechanisms through which risk factors might influence CRC development and highlight the potential chemopreventive roles of metformin and antihypertensive drugs in CRC risk.

## 1. Introduction

Colorectal cancer (CRC) is the third most diagnosed cancer and the second leading cause of cancer death affecting both men and women [1]. There is evidence that genetic factors are involved in CRC development, as revealed by the family history-associated risk and low penetrance genetic risk variants [2,3,4]. 

The incidence of CRC is also affected by modifiable lifestyle factors. Physical activity, alcohol consumption, smoking habits and adherence to a Western dietary pattern are among the key lifestyle factors linked to CRC risk [5]. Additionally, medication intake has been related to CRC. Notably, aspirin has been shown to reduce CRC risk by 20% [6,7], although conflicting results have been reported on this topic [8]. Beyond aspirin, non-steroidal anti-inflammatory drugs (NSAIDs) have also been linked to a decreased CRC risk [6,7,9,10]. However, their substantial gastrointestinal and cardiovascular side effects limit their use as chemopreventive agents [7]. Other medications explored for their potential to reduce CRC risk include the blood lowering glycemic agent metformin [9,11,12,13], the blood lipid reducing statins [14,15,16] and the antiresorptive bisphosphonates [17,18].

Modifiable factors and medications may influence CRC risk by altering cellular molecular mechanism. The various cell populations surrounding and within the tumor tissue are collectively referred to as the tumor microenvironment. Although these cells may appear to form normal tissue, molecular alterations, known as the cancer field effect (FE), have been observed [19,20,21]. The FE could either be a consequence of the presence of the tumor or indicate a pre-neoplastic phase of tumor development. The working hypothesis of this study is that, in the latter scenario, FE molecular changes could be the result of the mechanistic impact of risk exposures on the gut epithelium. Among these molecular mechanisms, gene regulation emerges as a pivotal player [22]. Notably, it has been observed that hundreds of genes were overexpressed in healthy colon tissue adjacent to tumors in comparison to tissue from healthy individuals. This gene expression drift was interpreted as part of the cancer FE within the tumor microenvironment [19,20].

Medications have the potential to modulate gene expression. Specifically, in the case of oral medication, the absorption through the intestinal barrier takes place in different locations of the gastrointestinal tract, mainly in the stomach and small bowel, but also in the colon [23]. Therefore, active ingredients and their metabolites have the potential to modify gut epithelial gene expression patterns, exerting either beneficial or harmful effects. Thus, the oral administration of medication could play a central or adjuvant role in the prevention and treatment of CRC by reversing gene expression patterns characteristics of the tumor microenvironment. The main limitation in assessing the impact of medication intake on colon tissue gene expression in an observational setting lies in the presence of non-controlled confounding effects from other factors or comorbid diseases. Moreover, conducting a randomized controlled trial (RCT) to assess this could raise ethical concerns. 

Genetic epidemiology can circumvent some of the inherent limitations of observational epidemiology by modeling non-genetic risk factors using genetic instruments and by evaluating their association with the outcome rather than between the modifiable factor and the outcome. In this study, we considered the use of allele scores as an instrumental variable. They are important for the modelling of multifactorial polygenic traits, particularly when the allele score consists of common variants with small effects [24]. Under the assumption that the genetic instruments used are specifically associated with the risk factor of interest, a genetic instrumental variable divides the population into subgroups that systematically differ in the risk factor, but not in any confounding factors. Germline gene variants related to a given risk factor are less prone to be associated with confounding exposures. The genetically defined subgroups are analogous to treatment arms in a RCT. Any difference in the outcome between the subgroups is inferred to be causally due to the risk factor of interest. Therefore, this technique, commonly referred to as Mendelian randomization (MR), is considered less sensitive to some of the biases that afflict traditional observational epidemiology, in particular reverse causality and residual confounding [25]. With the increased availability of the results of genome-wide association studies (GWASs), genetic variants in many loci have been identified for different complex traits, for instance obesity, smoking and alcohol intake. In the case of medication intake, genetically proxied therapeutic inhibition of a given drug target can be used to assess the effect of a drug on the outcome [26]. For instance, genetic variants in genes strongly related to systolic blood pressure, and inhibited by hypertensive drugs, have been used to evaluate the effect of hypertensive drugs on CRC risk [27]. 

The aim of this study was to investigate the impact of medication intake and modifiable factors on the expression of genes associated with cancer FE in the colon. This was performed using a dataset of 445 colon tissue samples from healthy Spanish individuals (BarcUVa-Seq: University of Barcelona and University of Virginia RNA sequencing project) [28,29]. Additionally, two independent datasets were incorporated, comprising 302 colon tissue samples (CEDAR: Correlated Expression and Disease Association Research) [30] and 386 colon tissue samples (GTEx: Genotype—Tissue Expression v8) [31]. In our MR methodology, we focused on testing variation in gene expression patterns rather than outcome risk. To achieve this, we used allele scores as instrumental variables within an individual-level RNA-Seq analysis framework. This individual-level framework is the foundation of the original MR methodology and yields results identical to those obtained from subsequent summary-based methods [32]. The results offer insights into the biological mechanisms by which medication intake and risk factors may contribute to reduce or increase CRC risk. To the best of our knowledge, this is the first study evaluating the effect of common medication intake and exposures on gene expression patterns related to the tumor microenvironment in healthy colon mucosa using both observational and instrumental approaches. 

## 2. Results

### 2.1. Field Effect Gene Signature 

A robust collection of 211 genes was identified as an FE signature using two previous studies independent of the BarcUVa-Seq project [19,20]. These FE genes were upregulated in healthy colon tissue adjacent to tumor compared to healthy tissue from healthy individuals (Supplementary Table S1). In the BarcUVa-Seq dataset, the overall expression of the FE genes was lower than the expression of the rest of the genes (Wilcoxon *p*-value = 5.74 × 10^−11^, Supplementary Figure S1). Pathway analysis of the 211 genes showed enrichment in extracellular matrix-related functions, muscle contraction and signaling. Supplementary Table S2 and Supplementary Figure S2 show all seven significantly enriched pathways. Of note, 51 out of the 211 selected genes (chi-squared *p*-value = 2.2 × S10^−81^) were also present in a recent colon FE study that included the BarcUVa-Seq dataset in the analyses [21].

### 2.2. Sample Description: Clinical Parameters and Medication Intake 

Some differences were seen in age distribution and gender proportion for the three tested datasets. The BarcUVa-Seq subjects (64% females) had a mean age of 60 years and were mostly of European ancestry. The CEDAR dataset comprised 56% women and had a mean age of 55 years. Finally, the GTEx dataset had a lower proportion of women (37%) and the ages ranged between 20 and 80 years, although most of the participants (35%) were between 50 and 59 years. While genetic and gene expression data were available for the three datasets, only the BarcUVa-Seq and CEDAR datasets had data on body mass index (BMI) and smoking status. Mean BMI was similar for both datasets (27.5 ± 4.2 and 26.2 ± 4.6 for BarcUVa-Seq and CEDAR, respectively). Smoking status indicated that 13.7% of the individuals in the BarcUVa-Seq dataset were current smokers, 33.8% were former smokers and 52.5% had never smoked. In the CEDAR dataset, 20.5% of the individuals were current smokers, while 79.5% were either former smokers or had never smoked. In addition, 69.9% and 20.6% of the BarcUVa-Seq participants were non-drinking women and men, respectively; while among the drinkers, the median was 2.2 g/day among women and 5.1 g/day among men. Finally, the BarcUVa-Seq dataset also registered overall physical activity (30.4± 26.8 units of metabolic equivalent task hours per week (MET-h/w units of metabolic equivalents per week (METS)) (Supplementary Table S3). 

Finally, only the BarcUVa-Seq dataset had data on both clinical outcomes and medication intake. The individuals in the BarcUVa-Seq dataset may have been diagnosed with prevalent diseases typically found among people within their age range (39–80). These clinical outcomes were equally distributed among the sexes except for osteoporosis and depression, which were more common among females, and gout disease, which was more prevalent among men (*p*-value < 0.01; Table 1). Of note, the individuals ever diagnosed with cancer (4%) included survivors of non-digestive cancers who had overcome the disease. The intakes of a wide range of medications were associated with clinical outcomes. Some of the medications most significantly associated with the clinical outcomes were calcium supplements for osteoporosis (*p*-value = 2.77 × 10^−18^); metformin for type 2 diabetes (*p*-value < 1.00 × 10^−20^); antihypertensive medications for hypertension (*p*-value < 9.58 × 10^−5^); statins for hypercholesterolemia (HMG CoA reductase inhibitors: *p*-value < 1.00 × 10^−20^); and benzodiazepine derivatives and antidepressants for major depressive disorder (Table 1 and Supplementary Table S3). As far as the relationship between modifiable exposures and medication intake is concerned, BMI was associated with the intake of omeprazole, hypertensive drugs, antidepressants and respiratory system drugs (*p*-value < 0.01; Supplementary Table S3). Drinking status and smoking status were not associated with medication intake (Supplementary Table S3).

### 2.3. Gene Expression Patterns by Medication Intake in the Observational Setting

**Figure 1:**
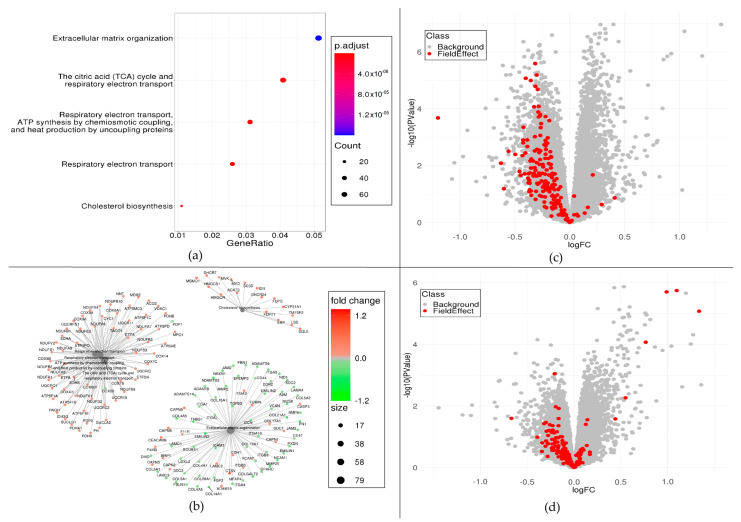
(**a**) Reactome-based enrichment analysis summary for metformin intake. Dots, proportional to the number of genes associated with specific pathways, represent the most significantly enriched pathways and are color-coded based on adjusted *p*-values. (**b**) Network plot representing the significant genes found for the most significant pathways. Genes are colored by the fold change in metformin intake profile. (**c**) Metformin volcano plot. Differential gene expression for metformin intake. (**d**) rs17485664 volcano plot. Differential gene expression for rs17485664 profile. For (**c**,**d**), *x*-axis represents logFC values, *y*-axis represents −log10 *p*-values, horizontal dashed red line represents significant adjusted *p*-value threshold (FDR = 0.1), red dots represent FE genes and gray dots the rest of background genes.

Differential expression analysis (DEA) was applied to the medication intake variables in the BarcUVa-Seq dataset adjusting for sex, age, tissue location and RNA sequencing batch. We observed that the individuals treated with biguanides used to treat type 2 diabetes (metformin—A10BA02) showed a total of 1364 upregulated genes and 1350 downregulated genes compared to those without treatment (Table 2). Functional analyses of these 2714 genes showed an enrichment of 21 pathways (FDR < 0.05), most of them related to respiratory electron transport, steroid metabolism and extracellular matrix organization (Figure 1a,b and Supplementary Table S4). Up- and downregulated genes can be observed as scattered dots in Figure 1c, depicting the significance of the DEA association estimates (logFC). It is worth mentioning that the participants under metformin treatment showed an overexpression of almost all the genes present in cholesterol biosynthesis (17 out of 25, see Figure 1b). Other drugs showed lower numbers of differentially expressed genes, such as platelet aggregation inhibitors (acetylsalicylic acid, 42 genes), diuretic drugs (hydrochlorothiazide, 27 genes) and lipid modifying agents (statins, 18 genes) (Table 2). 

We compared the distributions of the DEA association estimates of the 211 FE genes with the overall genes using Gene Set Enrichment Analysis (GSEA) [33]. Under the assumption that the upregulation of the FE genes is promoting tumor development, each analyzed parameter was considered as a risk or protective factor depending on whether the FE genes showed a positive or negative enrichment score (ES), respectively, in the GSEA. A total of 16 medications appeared to attenuate the expression of the FE genes as they showed a negative ES (*p*-value < 0.01) (Table 2). The medications showing the strongest negative ES were metformin (ES = −2.95; *p*-value = 1.18 × 10^−3^) (Figure 1c, red dots of FE genes were mostly downregulated compared with the background genes in grey), paracetamol (ES = −2.91; *p*-value = 1.93 × 10^−3^), antihypertensives (ES = −2.84; *p*-value = 1.29 × 10^−3^), statins (ES = −2.82; *p*-value = 2.29 × 10^−3^) and anti-inflammatory and antirheumatic products (ES = −2.82; *p*-value = 1.39 × 10^−3^). By contrast, it was observed that calcium supplement intake promoted the upregulation of the FE genes as it showed a mild positive ES (ES = 1.62; *p*-value = 4.64 × 10^−3^), while acetylsalicylic acid intake also showed a mild positive but non-significant ES (ES = 1.40; *p*-value = 0.012) (Table 2). 

### 2.4. Gene Expression Patterns by Medication Intake in the Instrumental Setting

To estimate the effect of medication intake on gene expression under an instrumental (MR) procedure, we applied DEA and GSEA analyses using as instrumental variables allele dosages of SNPs mimicking the effect of the medication. The top expression quantitative trait locus (eQTL) for each drug target was identified as genetic proxies of drug action. Robust genetic proxies (Fstat ≥ 10) for the therapeutic response of drug targets were found for the inducer effects of metformin on *PRKAB1* (rs17485664), calcium supplements on *CACNA1C* and *ATP2C1* (rs78155648 and rs112703671, respectively) and diazepam on *GABRA2* (rs1442060), and for the inhibitor effects of amlodipine on *CACNA1C* (rs78155648) and enalapril on *ACE* (rs4292) (Supplementary Table S5). It is worth noting that the proxy rs78155648 for *CACNA1C* was used as an instrument for both the inductive effect of calcium supplements and the inhibitor effect of amlodipine (calcium channel blocker). 

The results showed that genetic proxies provided similar enrichment patterns of expression (ES) to the corresponding medications (Table 3). According to these instrumental results, the inductive effect of metformin on *PRKAB1* provided an underexpression of the FE genes (ES = −1.86, *p*-value = 2.33 × 10^−3^) (Figure 1d, red dots represent FE genes that were mostly downregulated compared with the background genes, shown in grey), which was validated in the other two datasets (ES = −1.39, *p*-value = 4.00 × 10^−3^, ES = −2.00, *p*-value = 1.06 × 10^−3^, in GTEx and CEDAR, respectively). The inducer effect of calcium supplements on *CACNA1C* and *ATP2C1* showed a trend to increase the expression of the FE genes (ES = 1.51, *p*-value = 0.08, and ES = 2.03, *p*-value = 2.23 × 10^−3^, respectively), which was not fully validated in the case of *CACNA1C* in the other datasets (ES = 1.25, *p*-value = 0.07, in GTEx; and ES = 1.66, *p*-value = 2.31 × 10^−3^, in CEDAR), nor validated in *ATP2C1* (ES = 2.57, *p*-value = 1.04 × 10^−3^, in GTEx; and ES = −1.83, *p*-value = 0.01, in CEDAR). Therefore, the inhibitor effect of calcium channel blocker (amlodipine) on *CACNA1C* showed a trend to underexpress the FE genes (same statistics but in opposite direction to the inducer effect of calcium supplements). This effect of the antihypertensive drug was also observed in the case of the agents acting on the renin–angiotensin system. The inhibitor effect of enalapril on *ACE* exhibited an underexpression of the FE genes (ES = −2.00, *p*-value = 1.60 × 10^−3^), which was validated in GTEx (ES = −2.22, *p*-value = 2.84 × 10^−3^) and in CEDAR, although non-significantly (ES = −1.05, *p*-value = 0.33). 

Finally, the inductive effect of diazepam on GABRA2 showed a remarkable overexpression of the FE genes (ES = 2.33, *p*-value = 1.88 × 10^−3^), which was confirmed in both GTEx and CEDAR (ES = 2.29, *p*-value = 1.66 × 10^−3^, ES = 2.45, *p*-value = 2.07 × 10^−3^, respectively).

### 2.5. Gene Expression Patterns by Modifiable Exposures in the Observational Setting

The differential expression results in the BarcUVa-Seq dataset indicated that each standard deviation (SD) increase in BMI was related to the upregulation of 34 genes and the downregulation of 22 genes (Table 4). The genes in the highest quartile of mean expression values in both the BarcUVa-Seq and CEDAR DEA results showed a positive FC correlation (n = 1776 genes, Spearman’s r = 0.27, *p*-value = 1.43 × 10^−31^). Both datasets showed a negative ES for the 211 FE genes (−2.71, *p*-value = 0.001 in BarcUVa-Seq; and −2.32, *p*-value = 0.004 in CEDAR) (Table 4). Regarding smoking status, the genes in the highest quartile of mean expression values in both datasets showed a moderate positive correlation (Spearman’s r = 0.19, *p*-value = 3.56 × 10^−16^) for DEA between current smokers and individuals who had stopped smoking or never smoked. A positive ES for smoking was observed in BarcUVa-Seq (1.4, *p*-value = 0.04) and in CEDAR (0.79, *p*-value = 0.90, but non-significant) (Table 4). In the case of drinking habits, the observational results indicated that individuals with a higher intake of alcohol showed a total of 587 upregulated genes and 568 downregulated genes compared with individuals with a lower alcohol intake. A negative ES was observed in BarcUVa-Seq (−1.66, *p*-value = 0.001) (Table 4). Finally, overall physical activity showed a positive ES (1.44, *p*-value = 0.009) (Table 4).

### 2.6. Gene Expression Patterns by Modifiable Exposures in the Instrumental Setting

In the instrumental analyses, we used weighted genetic scores as instrumental variables for DEA and GSEA analyses in a two-sample individual-level MR approach. Genetic proxies for modifiable exposures (BMI, smoking initiation, drinks/week) were identified in large GWASs of the risk factors of interest, and these genetic proxies were tested for association in the three gene expression datasets. In the case of BMI, in contrast to the observational results, the instrumental analyses showed a positive ES in BarcUVa-Seq and GTEx (2.47, *p*-value = 0.003; 2.98, *p*-value = 0.006) but a negative trend in CEDAR (−1.38, *p*-value = 0.02) (Table 4). Regarding smoking status, a positive ES was observed in BarcUVa-Seq, GTEx and CEDAR (2.45, *p*-value = 0.003, 3.45, *p*-value = 0.006, and 0.91, *p*-value = 0.71, respectively) (Table 4). Finally, for drinking habits, a negative ES was observed in BarcUVa-Seq (−2.65, *p*-value = 0.002), but the results in CEDAR and GTEx showed a positive ES (1.53, *p*-value = 0.10 and 2.77, *p*-value = 0.002) (Table 4).

## 3. Discussion

In this article, a potential mechanism of the chemopreventive effect on CRC risk of metformin and antihypertensive agents acting on the renin–angiotensin system was identified. Also, we identified a potential mechanism of the observed harmful effect of benzodiazepine derivatives on increasing CRC risk. This was achieved by analyzing the differential expression in colon mucosa of healthy individuals of a set of 211 genes related to the cancer field effect. This analysis was performed using both observational and instrumental approaches with replication of the results, which provided robust support for the findings.

### 3.1. Gene Expression in the Tumor Microenvironment

The molecular alterations (field effect—FE) present in the cell populations surrounding the tumor cells (the tumor microenvironment) are considered necessary for promoting the growth and survival of cancer cells. To characterize the colon cancer FE at gene expression level, we proposed a robust set of genes from two different studies independent of the BarcUVa-Seq project [19,20]. The pathway analysis of these genes is in accordance with changes in the composition of the extracellular matrix and the dysregulation of the tissue homeostasis, which contribute to both the development and progression of neoplastic lesions [34,35,36]. Furthermore, the lower expression values of these genes observed in the healthy colon samples could indicate that FE genes may play a role in the early pre-neoplastic stage of tumor development.

### 3.2. Chemopreventive Role of Commonly Used Medication

The observational and instrumental results for medication intake were highly concordant. Metformin was identified as the medication with the highest impact on gene expression, altering the respiratory electron transport, the steroid metabolism, the extracellular matrix organization and the cholesterol biosynthesis. In addition, metformin showed the strongest enrichment in downregulating the genes associated with cancer FE, which was validated in the instrumental analyses including three independent datasets. These results are in accordance with the theoretical anticancer effects of metformin, which are divided into direct effects, reducing the energy consumption of cancer cells by inhibiting mitochondrial respiratory chain complex I, and indirect effects, reducing fasting plasma insulin levels [37,38]. This evidence provides a solid mechanism of action for the observed lower associated CRC risk to patients with diabetes under metformin treatment.

Our results are consistent with a chemoprotective effect of *ACE* inhibitors on CRC risk. Recent observational results on the use of renin–angiotensin system inhibitors pointed to a protective effect or null effect of these anti-hypertensive drugs on CRC risk [39,40,41]. Also, immunosuppression roles have been proposed for *ACE* in the tumor microenvironment, which provide a potential benefit of *ACE* inhibition in the development of neoplasm [42]. On the other hand, a previous MR study mimicked the *ACE* inhibitor effect using genetic variants of the *ACE* region associated with systolic blood pressure (SBP) [27]. They found a risk role of *ACE* inhibition not mediated by SBP. However, they also found that genetically proxied *ACE* gene expression was positively associated with CRC risk. Therefore, in line with these previous results, our results support a chemopreventive effect of *ACE* inhibition mediated by *ACE* gene expression and downregulating cancer FE genes. On the other hand, the intake of calcium supplements showed upregulation of the cancer FE genes, but this was not fully validated by the instrumental analyses. These results are in sharp contrast with the previously observed relation between supplementary calcium intake and CRC risk. It has been observed how calcium intake showed a protective role for CRC risk in prospective and case–control studies [43,44]. Also, serum calcium concentrations were observed to be inversely associated with the risk of CRC [45]. In addition, in a study of patient-derived organoids, the exposure of organoids to concentrations of calcium for 72 h revealed alteration of the gene expression of CRC-related genes, which could explain the chemopreventive role of calcium in CRC risk [46]. A potential explanation for our results could be that the chemopreventive effect of calcium is not mediated by the expression of the *CACNA1C* or *ATP2C1* genes. A recent study analyzing genetic variants in the calcium signaling pathway pointed to the *PDE1C* gene as a key gene contributing to CRC through changes in the tumor microenvironment [47]. In addition, our instrumental results could be confounded as the *CACNA1C* gene is also targeted by anti-hypertensive calcium channel blockers. 

Regarding our results related to the use of benzodiazepine derivatives, a non-significant downregulation of cancer FE genes was observed. However, our instrumental results provided the opposite effect, a strong upregulation of FE genes, indicating that the observational results could be confounded. Previous observational case–control and cohort-based studies indicated that the use of benzodiazepines increased the risk of specific cancers, including CRC in 5–25% [48,49,50]. The mechanisms involved in this risk association remain unclear and controversial. However, they could involve chronic inflammation and tumor growth [47,49]. Therefore, our study supports these previous observations and provides a potential mechanism of action of the role of benzodiazepines in the CRC risk.

In summary, our results reinforce the observed beneficial effect of known drugs in reducing CRC risk [39,51,52] and provide a potential mechanism of action modulating cancer FE gene expression in the tumor microenvironment. They provide new potential therapeutic strategies for CRC, since oral drug administration is a common delivery route for a wide range of therapies and is strongly preferred by patients for its ease of use due to its inherent non-invasiveness [53,54]. 

### 3.3. The Contribution of Modifiable Risk Factors to the Tumor Microenvironment

The observational and instrumental results for modifiable risk factors showed some discrepancies. They highlighted the possibility of unmeasured confounding affecting the observational results. In fact, higher BMI was associated with the intake of proton-pump inhibitors, antihypertensive drugs, antidepressants and respiratory system drugs. The independent genetic polymorphisms included in the instruments were randomly allocated at conception and cannot be correlated with potential confounders in a similar way [24]. The instrumental findings of this study in relation to smoking parameters, and to a lesser extent to BMI, raise the possibility of a potential causal relationship between these factors and the observed alterations in gene expression. The causes of the molecular alterations of the tumor microenvironment that benefit cancer cells are not well understood. On one hand, it has been observed that cancer cells release growth factors and cytokines in the microenvironment [55]. However, this study supported the idea that it can be also the result of the effect of CRC risk exposures on the gut epithelium. The validated genetic instruments provided strong positive associations in BarcUVa-Seq and GTEx, but these results were not robustly validated in the independent CEDAR dataset. This independent dataset was composed of slightly younger people (mean age of 55 years versus 60 years), which could reduce the required cumulative period of exposure to have a significant impact on health. Therefore, replication results in other datasets composed of older people are encouraged. Other potential sources of bias in this instrumental setting are the presence of pleiotropic effects and measurement errors [24]. However, the large number of genetic instruments used, the selection of the largest sample size studies used to identify them, the analysis based on gene set enrichment instead of a single gene and the internal validation performed in our study minimized these sources of bias.

### 3.4. Strengths and Limitations 

To the best of our knowledge, we have analyzed the largest cross-sectional dataset that includes anthropometric, lifestyle and clinical data of participants together with germ-line genetics and gene expression data of healthy colonic mucosa. There exist similar studies with a smaller number of participants, addressing diet or lifestyle factors [56,57], and studies with similar genetic and gene expression data but without lifestyle and clinical data [31]. 

This study has several limitations. As main limitation, in this study there were some medication effects that could not be tested in the instrumental setting because no strong genetic instruments at gene expression level were identified. This was the case for omeprazole, lipid-lowering drugs, antidepressants, acetylsalicylic acid and NSAIDs. In addition, acetylsalicylic acid and NSAIDs share common target *PTGS1* and *PTGS2* genes, which enables disentanglement of the effects of these drugs in an instrumental setting. Larger studies to identify robust genetic instruments are needed to further investigate these drugs. In addition, some of the analyzed diseases and their associated treatments were supported by a small fraction of the whole dataset, which could reduce the robustness of the results. Observational variables may have minimal biases even though qualified professionals conducted the interviews and posterior quality control was carried out to remove outliers or contradictory answers. Regarding the gene expression data, bulk RNA-seq is a mixture of different cell compositions; thus, the gene expression levels reflect weighted average expression levels based on the number and diversity of cell types. However, these minimal biases are unlikely to affect our results. Another inherent limitation of MR studies is that they can be slightly biased due to uncontrolled confounding from family effects such as assortative mating, dynastic effects and population structure [58]. Although we mitigated bias from population stratification using a two-sample MR analysis in European ancestry samples, our findings may not be applicable to populations of non-European ancestry.

## 4. Materials and Methods

### 4.1. BarcUVa-Seq Dataset 

The BarcUVa-Seq (University of Barcelona and University of Virginia RNA sequencing project) dataset is a cross-sectional study including bulk RNA sequencing data from colon mucosa and blood genome-wide germline data of 445 healthy adult donors interviewed for diet, lifestyle and medical variables [28]. The modifiable exposures comprise BMI, smoking status (current smoking, no smoking), overall physical activity (registered in units of metabolic equivalent task hours per week (MET-h/w)) and alcohol intake (measured as described elsewhere) [59]. The continuous parameter alcohol intake (registered in grams per day) was categorized by sex in three ordinal categories: non-drinkers, moderate drinkers and heavy drinkers. Non-drinkers were considered the reference category, while moderate and heavy drinkers were identified below and above the median within sex among drinkers. In addition, diagnostics for clinical outcomes (defined using International Classification of Diseases codes, version 10 (ICD-10)) [60] and medication intake (defined using Anatomical Therapeutic Chemical code (ATC)) [61] were also included in the analyses. A total of 428 individuals had both questionnaire and clinical data available. Biopsies were obtained from sites along the ascending (n = 135; 31.5%), transverse (n = 140; 32.7%) and descending (n = 153; 35.8%) colon, from one site per individual. Molecular profiling was previously described in [28]. Briefly, the RNA extraction, quantification and sequencing of colon samples were performed using Illumina HiSeq 2500 or NovaSeq 6000 instruments. The filtering steps for the expression data consisted of the removal of genes smaller than 300 bp or with less than one Count Per Million (CPM) in at most 100 samples. This was done to exclude miRNA from the final expression matrix, as minimal miRNA captured by the protocol could lead to biased analysis for this RNA typology. Protein coding and non-coding gene expression levels were then normalized using the Trimmed Mean of M values (TMM) method. This ensured optimal results for the differential expression analysis and a robust comparison between the samples with adjustment by library size [62]. Principal Component Analysis (PCA) was performed to inspect for possible outliers and biases of common nature, such as sex, age and batch processing. Blood DNA genotyping was performed with Illumina OncoArray BeadChip and imputed, resulting in 6,804,675 single nucleotide polymorphisms (SNPs) with an imputation R^2^ > 0.7 and a minor allele frequency (MAF) > 1%. This dataset is representative of the transcriptome of colon epithelial cells of living subjects, as all the biopsies were collected from superficial mucosa at colonoscopy. This characteristic makes it optimal for investigating the normal physiology across the colon, and it is relevant for studying etiological aspects of diseases affecting this tissue. More information about the BarcUVa-Seq project can be accessed online at https://barcuvaseq.org/, accessed on 27 June 2024. In addition, the transcriptomic features of this dataset and their association with germline genetic variants can be explored online in a web browser, the Colon Transcriptome Explorer version 2.0 [29] (CoTrEx 2.0; https://barcuvaseq.org/cotrex/, accessed on 27 June 2024). 

### 4.2. CEDAR Dataset 

The CEDAR (Correlated Expression and Disease Association Research) dataset [30] was used as an independent dataset for validation purposes. The epidemiological parameters available in this dataset were BMI and smoking status (current vs. non-smokers). Therefore, in this dataset, we tested the effect of BMI and smoking status in both the observational and instrumental setting, and the effect of the intake of drugs in the instrumental setting. The CEDAR dataset was obtained from the Array Express repository under accession numbers E-MTAB-6666 and E-MTAB-6667 for the genotypes and expression data, respectively. The data used in this study included gene expression from the transverse colon (302 individuals). Quality control for genotyping and expression data was previously described elsewhere [63]. Briefly, the microarray raw expression data was normalized using negative control probes with the limma R package [64], while the genotypes were imputed using the Haplotype Reference Consortium panel on the Michigan Imputation Server, resulting in 7,374,172 SNPs with an imputation R^2^ > 0.7 and a MAF > 1%. 

### 4.3. GTEx Dataset 

The GTEx (Genotype—Tissue Expression) dataset v8 [31] was also used as an independent dataset for validation purposes for the effect of medication intake in the instrumental setting. Sex and age were the epidemiological parameters available in this dataset. The transverse colon GTEx v8 dataset comprised 368 samples and was obtained from dbGaP (study accession: phs000424.v8.p2). The raw expression count data were analyzed using exactly the same procedure as described before for BarcUVa-Seq. 

### 4.4. Gene Set of Cancer FE 

A robust collection of genes characterizing the cancer FE of colonic mucosa was built using two previous studies [19,20]. The selected genes showed differential expression in both studies between healthy tissue adjacent to tumor and colon tissue samples from healthy individuals. The selected genes showed log2 fold change (logFC) values in the same direction, absolute logFC values greater than 1 and Bonferroni correction *p*-values lower than 0.01. A total of 222 genes were selected and split into 211 upregulated genes and 11 downregulated genes (Supplementary Table S1). For the sake of statistical robustness, only the set of upregulated genes were selected for subsequent analyses. The Wilcoxon rank sum test was employed to evaluate differences in the expression values among FE genes and the remaining expression profile in the BarcUVa-Seq samples. Pathway analysis was performed for the selected genes with ReactomePA [65] R package. Pathways with a Benjamini–Hochberg false discovery rate (FDR) lower than 0.05 were considered significant. 

### 4.5. Observational Analyses 

Differential expression analysis (DEA) was applied to the standardized and categorical variables. The Generalized Linear Model (GLM) with a quasi-likelihood method was used to ensure a robust error rate control. Models were adjusted by sex, age, tissue location and RNA sequencing batch. A Benjamini–Hochberg false discovery rate (FDR) of 0.1 was set to select statistically significant differential expressed genes. As result, the logFC estimate expresses 2-fold increase in the levels of gene expression per standard deviation (SD) of increase for the continuous variables, and per category compared with the reference category for the categorical variables. All the filtering and statistical analyses were performed with the edgeR R package [66]. The logFC distribution of the 211 FE genes compared with the logFC distribution of overall genes was assessed by Gene Set Enrichment Analysis (GSEA) [33] for each variable. Under the assumption that FE drift indicates a pre-neoplastic phase of tumor development, each analyzed parameter was considered as a risk or protective factor depending on whether the FE genes showed a positive or negative enrichment score (ES), respectively. GSEA results with a *p*-value smaller than 0.01 were considered statistically significant. For the medication parameters, analyses were performed for those variables that had at least 5% of the sample (20 individuals) in one of the two categories, in order to ensure there were enough samples in both groups. 

### 4.6. Instrumental Analyses 

To estimate the effect of medication intake on the whole gene expression under an instrumental procedure, we considered a two-sample individual-level MR approach using as instrumental variables allele dosages of SNPs mimicking the effect of the medication. The DrugBank database (https://go.drugbank.com/, accessed on 27 June 2024) [67] was used to identify genes targeted by drugs. Subsequently, as genetic proxies of drug action, the top expression quantitative trait locus (eQTL) for each drug target was identified in the BarcUVa-Seq dataset and validated in the GTEx and CEDAR datasets (*p*-value < 0.01). Only eQTLs validated in at least one of the two datasets were considered for further analyses. The “relevance” assumption was also tested by estimating the proportion of variance of gene expression in the BarcUVa-Seq dataset explained by the eQTLs (as gene expression levels were transformed using an inverse normalization) and estimating the F-statistics. F-statistics can be used to estimate the strength of the relationship between the genetic instrument and the proxied exposure, which is an estimation of the magnitude of the instrument bias (e.g., Fstat < 10 for the weak instruments). Genotypes were recoded to 0, 1 and 2 as an additive genetic model according to the number of carried alleles mimicking the drug effect, i.e., decreasing gene expression when mimicking an inhibitor or an antagonist, or increasing gene expression when mimicking an inducer, an agonist or a ligand. 

To estimate the effect of the modifiable exposures on the whole gene expression under an instrumental procedure, we considered a two-sample MR approach using weighted genetic scores as instrumental variables in the individual-level gene expression datasets. A weighted gene score is a single variable summarizing multiple independent genetic variants associated with a risk factor, calculated as the sum of weights for the total number of risk factor-increasing alleles for an individual, being the weights of the estimated genetic effect size for the exposure in the discovery study (βSNP-to-trait). Genetic proxies for modifiable exposures (BMI, smoking initiation, drinks/week, overall activity) were identified in several large GWASs of the risk factors of interest (as the first set of samples), and these genetic proxies were tested for association in the previously described gene expression datasets (as the second set of samples) [28,30,31]. 

Under MR assumptions, genetic scores avoid weak instrument bias and enable valid causal estimates with large numbers of genetic variants [24]. The MR approach assumes that the used genetic instruments are specifically associated with the risk factor of interest and are not directly associated with either the outcome or any potential confounding variable. Violation of the assumptions can occur in front of pleiotropic association of the genetic variants in linkage disequilibrium (LD) with another functional variant, and in front of population stratification where genetic associations reflect latent strata in the population. To avoid violations of MR assumptions, the genetic instruments for each risk factor were SNPs independently (LD R2 measure < 0.01) associated with the trait at a genome-wide level (*p* < 5 × 10^−8^) identified in the most recent and largest GWAS results on that trait from samples of European ethnicity. 

Results from the Genetic Investigation of ANthropometric Traits (GIANT) consortium and the UK Biobank were used to identify genetic proxies for body mass index (BMI) [68]. Similarly, data from the Tobacco and Genetics (TAG) consortium were used to identify genetic loci for smoking initiation and average alcohol intake, registered as drinks per week [69]. Finally, genetic instruments for overall physical activity were identified from a genetic study on the UK Biobank [70]. For each identified SNP, the reported effect allele size was for the allele associated with an increase in the trait and expressed in one standard deviation (SD) of the trait per allele (βSNP-to-trait). SNPs with ambiguous strand codification (A/T or C/G) were replaced by SNPs with tight genetic linkage (LD R^2^ > 0.8) in European populations using the SNP Annotation and Proxy Search (SNAP) (https://www.broadinstitute.org/mpg/snap/ldsearch.php, accessed on 2 July 2024) or removed from the analyses. Finally, the “relevance” assumption was tested by estimating the proportion of variance explained for each risk factor and by estimating the F-statistics. The number of identified SNPs, proportion of variance explained for each risk factor and the F-statistics are detailed in (Supplementary Table S6).

In addition, to further assess the validity of the genetic instruments, genetic scores were evaluated for their association with both proxy exposures and potential confounding factors present in the dataset. These relationships were modeled using linear regression, controlling for age, sex and principal components to account for population stratification. One standard deviation (SD) increase in genetically determined BMI was strongly associated with standardized BMI in both datasets (beta estimate (est) = 0.47, *p*-value = 3.2 × 10^−3^, in BarcUVa-Seq; and est = 0.52, *p*-value = 7.5 × 10^−4^, in CEDAR). In addition, the BMI instrument was not associated with other potentially confounding factors, which confirms the validity of the instrument for BMI (Supplementary Figure S3; Supplementary Table S7). In the case of smoking, the genetic instrument for smoking status showed a positive, but non-significant, association with current smoking status (est = 0.81 for each standard deviation (SD) increase in the liability for smoking initiation, *p*-value = 0.25, in BarcUVa-Seq; and est = 0.24, *p*-value = 0.71, in CEDAR); of note, any association with the other exposures was not observed (Supplementary Figure S3; Supplementary Table S7). Regarding drinking status, the genetic instrument for alcohol intake was strongly associated with drinking status (est = 1.28, *p*-value = 1.6 × 10^−3^, in BarcUVa-Seq), especially with heavy drinking (est = 0.79, *p*-value = 2.2 × 10^−3^, in BarcUVa-Seq). This instrument was not associated with other potentially confounding factors, confirming its validity (Supplementary Figure S3; Supplementary Table S7). Finally, the genetic instrument for overall physical activity was not associated with measured overall physical activity, while it was inversely associated with drinking status (Supplementary Figure S3; Supplementary Table S7). Therefore, this instrument was not considered reliable and was not further used in this study. 

Differential expression analysis (DEA), the GLM and Gene Set Enrichment Analysis (GSEA) of the FE genes were applied for the genetically proxied medication intake and modifiable exposures in the BarcUVa-Seq, CEDAR and GTEx datasets following similar procedures to those used for the measured exposures. 

## 5. Conclusions

In this study, we identified a potential mechanism of the chemopreventive effect on CRC risk of metformin and antihypertensive agents acting on the renin–angiotensin system. The potential anti-cancer cell effect of metformin and antihypertensive agents suggests that they could be promising candidates for adjuvant therapy in cancer treatment. Nevertheless, further validation is required through clinical trials to confirm this possibility. In addition, we identified increased BMI and smoking as potential causal factors for the generation of a tumor-promoting microenvironment in the colonic mucosa, underscoring the importance of future research on lifestyle factors and their role in colorectal carcinogenesis.

## Figures and Tables

**Table 1:** Chronic disease distribution, gender proportion and associated medications in BarcUVa-Seq dataset.

Clinical Parameters	Overall		Females		Males		Psex	Associated Medication (Top *p*-Value per Medication)
	N	Yes (%)	N	Yes (%)	N	Yes (%)		
Heartburn	405	20.0	260	21.0	145	23.0	0.45	Omeprazole (*p* = 5.27 × 10^−12^)
Ulcer	409	4.0	261	3.0	148	11.0	0.05	
Hiatal hernia	409	9.0	261	9.0	148	13.0	0.99	
Helicobacter pylori	409	3.0	261	3.0	148	8.0	0.94	
Irregular bowel rhythm	408	14.0	260	17.0	148	13.0	0.02	
Constipation	428	9.0	273	11.0	155	5.0	0.04	Drugs for constipation (*p* = 1.10 × 10^−7^)
Osteoporosis	409	11.0	261	16.0	148	6.0	2.76 × 10^−4^	Calcium supplements (*p* = 2.77 × 10^−18^)
Diabetes	409	12.0	261	1.0	148	19.0	0.13	Metformin (*p* < 1.00 × 10^−20^)
Circulatory diseases	406	8.0	261	6.0	145	18.0	0.02	Acetylsalicylic acid (*p* = 3.39 × 10^−7^)
Anemia	408	6.0	260	7.0	148	8.0	0.14	
Hypertension	408	37.0	260	33.0	148	45.0	0.04	Hydrochlorothiazide (*p* = 9.58 × 10^−5^); Beta blocking agents, selective (*p* = 9.97 × 10^−7^); Atenolol (*p* < 1 × 10^−20^); Bisoprolol (*p* < 1 × 10^−20^); Amlodipine (*p* < 1 × 10^−20^); Enalapril (*p* < 1.00 × 10^−20^); Angiotensin II antagonists (*p* < 1.00 × 10^−20^)
Cholesterol	409	38.0	261	36.0	148	44.0	0.27	HMG CoA reductase inhibitors (*p* = 6.98 × 10^−10^)
Arthritis	408	22.0	261	26.0	147	2.0	0.03	Anti-inflammatory and antirheumatic products, non-steroids (*p* = 3.01 × 10^−9^); Ibuprofen (*p* = 3.19 × 10^−3^); Paracetamol (*p* = 8.84 × 10^−12^)
Thyroid disease	407	7.0	260	1.0	147	5.0	0.98	Levothyroxine sodium (*p* = 3.49 × 10^−9^)
Gout disease	408	5.0	261	2.0	147	15.0	5.84 × 10^−4^	
Depression	408	23.0	260	28.0	148	18.0	1.66 × 10^−3^	Benzodiazepine derivatives (*p* = 3.30 × 10^−14^); Antidepressants (*p* = 7.01 × 10^−17^); Selective serotonin reuptake inhibitors (*p* = 1.20 × 10^−8^);
Migraine	408	4.0	261	5.0	147	8.0	0.31	
COPD	409	8.0	261	8.0	148	12.0	0.9	Drugs for obstructive airway diseases (*p* = 2.24 × 10^−10^);
Cancer	409	4.0	261	4.0	148	8.0	0.86	
Renal lithiasis	407	4.0	260	3.0	147	12.0	0.06	
Bladder stones	408	2.0	260	2.0	148	8.0	0.37	

Psex: sex proportion test *p*-value; *p*: *p*-value; N: sample size; %: proportion.

**Table 2:** Observational results for effects of medication intake on gene expression.

Medication	Intake Category		DEA		GSEA	
	No	Yes	N Genes Upreg	N Genes Downreg	ES	*p*-Value
A02BC01: Omeprazol	277	122	4	2	−1.33	0.027
A06A: Drugs for constipation	377	22	1	0	1.38	0.026
A10BA02: Metformin	366	33	1364	1350	−2.95	1.18 × 10^−3^
A12A: Calcium supplements	353	46	0	0	1.62	4.64 × 10^−3^
B01AC06: Acetylsalicylic acid	366	33	35	7	1.40	0.0124
C03AA03: Hydrochlorothiazide	375	24	24	3	−2.30	4.07 × 10^−3^
C07AB: Beta blocking agents, selective	366	33	2	1	−2.84	1.29 × 10^−3^
C07AB07: Bisoprolol	376	23	5	0	−2.77	1.80 × 10^−3^
C08CA01: Amlodipine	375	24	1	0	−2.33	1.32 × 10^−3^
C09AA02: Enalapril	362	37	0	0	−2.21	1.19 × 10^−3^
C09CA: Angiotensin II antagonists, plain	368	31	0	1	−1.78	1.20 × 10^−3^
C10AA: HMG CoA reductase inhibitors	293	106	15	3	−2.82	2.29 × 10^−3^
C10AA01: Simvastatin	317	82	2	2	−2.74	1.95 × 10^−3^
H03AA01: Levothyroxine sodium	378	21	9	0	−2.05	1.59 × 10^−3^
M01A: Anti-inflammatory and antirheumatic products, non-steroids	282	117	0	0	−2.82	1.39 × 10^−3^
M01AE01: Ibuprofen	324	75	2	0	−1.94	3.90 × 10^−3^
N02BE01: Paracetamol	336	63	0	0	−2.91	1.93 × 10^−3^
N05BA: Benzodiazepine derivatives	351	48	1	0	−1.37	0.0272
N06A: Antidepressants	344	55	0	2	−2.02	1.06 × 10^−3^
N06AB: Selective serotonin reuptake inhibitors	368	31	1	0	−2.12	1.13 × 10^−3^
R03: Drugs for obstructive airway diseases	376	23	3	1	−2.52	1.14 × 10^−3^

N genes upreg: number of genes upregulated; N genes downreg: number of genes downregulated; DEA: Differential expression analysis; GSEA: Gene Set Enrichment Analysis; ES: GSEA normalized enrichment score.

**Table 3:** Instrumental results for drug genetic proxies on gene expression.

								BarcUVa-Seq	CEDAR	GTEx
Medication	Action	Gene	RSID	chr	pos	RA	AA	ES (*p*-Value)	ES (*p*-Value)	ES (*p*-Value)
A10BA02: Metformin	Inducer	*PRKAB1*	rs17485664	12	120043595	T	C	−1.86 (2.33 × 10^−3^)	−2.00 (1.06 × 10^−3^)	−1.39 (4.00 × 10^−3^)
A12A: Calcium	Ligand	*CACNA1C*	rs78155648	12	2376292	G	A	1.51 (0.08)	1.66 (2.31 × 10^−3^)	1.25 (0.07)
A12A: Calcium	Agonist	*ATP2C1*	rs112703671	3	130613634	G	C	2.03 (2.23 × 10^−3^)	−1.83 (0.01)	2.57 (1.04 × 10^−3^)
C08CA01: Amlodipine	Inhibitor	*CACNA1C*	rs78155648	12	2376292	A	G	−1.51 (0.08)	−1.66 (2.31 × 10^−3^)	−1.25 (0.07)
C09AA02: Enalapril	Inhibitor	*ACE*	rs4292	17	61554341	T	C	−2.00 (1.60 × 10^−3^)	−1.05 (0.33)	−2.22 (2.84 × 10^−3^)
N05BA01: Diazepam	Ligand	*GABRA2*	rs1442060	4	46366067	G	A	2.37 (1.88 × 10^−3^)	2.45 (2.07 × 10^−3^)	2.29 (1.66 × 10^−3^)

Action: drug mechanism on target gene; RSID: SNP ID; RA: reference allele; AA: alternative allele; ES: normalized enrichment score of Gene Set Enrichment Analysis.

**Table 4:** Phenotype distribution for modifiable exposures, observational and instrumental results on gene expression.

Parameters	BarcUVa-Seq			CEDAR			GTEx
	Phenotype Distribution	Obs	Inst	Phenotype Distribution	Obs	Inst	Inst
Categorical	N (% Currrent)	ES (*p*-Value)	ES (*p*-Value)	N (% Currrent)	ES (*p*-Value)	ES (*p*-Value)	ES (*p*-Value)
Smoking	409 (14%)	1.37 (0.04)	2.45 (2.6 × 10^−3^)	322 (21%)	0.79 (0.90)	0.91 (0.71)	3.45 (6.1 × 10^−3^)
Drinking	406 (48%)	−1.66 (1.3 × 10^−3^)	−2.65 (2.3 x10^−3^)	322 (-)	-	1.53 (0.10)	2.77 (2.3 × 10^−3^)
**Quantitative**	**N** **(mean ± stdev)**			**N** **(mean ± stdev)**			
BMI	393 (27.5 ± 4.2)	−2.71 (1.3 × 10^−3^)	2.47 (3.5 × 10^−3^)	316 (26.2 ± 4.6)	−2.32 (4.2 × 10^−3^)	−1.38 (0.02)	2.98 (5.9 × 10^−3^)
Physical activity (MET-h/w)	338 (30.4 ± 26.8)	1.44 (0.01)	-	-	-	-	

Obs: observational approach, Inst: instrumental approach, N: sample size, ES: enrichment score, stdev: standard deviation.

## Data Availability

The datasets generated and analyzed in our study are available on reasonable request from the corresponding author Victor Moreno (v.moreno@iconcologia.net).

## Supplementary Materials

The following supporting information can be downloaded at: https://www.mdpi.com/article/10.3390/ijms252111395/s1.

## References

[1] Sung H., Ferlay J., Siegel R.L., Laversanne M., Soerjomataram I., Jemal A., Bray F. (2021). Global Cancer Statistics 2020: GLOBOCAN Estimates of Incidence and Mortality Worldwide for 36 Cancers in 185 Countries. CA. Cancer J. Clin..

[2] Huyghe J.R., Bien S.A., Harrison T.A., Kang H.M., Chen S., Schmit S.L., Conti D.V., Qu C., Jeon J., Edlund C.K. (2019). Discovery of Common and Rare Genetic Risk Variants for Colorectal Cancer. Nat. Genet..

[3] Huyghe J.R., Harrison T.A., Bien S.A., Hampel H., Figueiredo J.C., Schmit S.L., Conti D.V., Chen S., Qu C., Lin Y. (2021). Genetic Architectures of Proximal and Distal Colorectal Cancer Are Partly Distinct. Gut.

[4] Jasperson K.W., Tuohy T.M., Neklason D.W., Burt R.W. (2010). Hereditary and Familial Colon Cancer. Gastroenterology.

[5] Friedenreich C.M., Ryder-Burbidge C., McNeil J. (2021). Physical Activity, Obesity and Sedentary Behavior in Cancer Etiology: Epidemiologic Evidence and Biologic Mechanisms. Mol. Oncol..

[6] Chapelle N., Martel M., Toes-Zoutendijk E., Barkun A.N., Bardou M. (2020). Recent Advances in Clinical Practice: Colorectal Cancer Chemoprevention in the Average-Risk Population. Gut.

[7] Liang P.S., Shaukat A., Crockett S.D. (2021). AGA Clinical Practice Update on Chemoprevention for Colorectal Neoplasia: Expert Review. Clin. Gastroenterol. Hepatol..

[8] Ma S., Han T., Sun C., Cheng C., Zhang H., Qu G., Bhan C., Yang H., Guo Z., Yan Y. (2021). Does Aspirin Reduce the Incidence, Recurrence, and Mortality of Colorectal Cancer? A Meta-Analysis of Randomized Clinical Trials. Int. J. Color. Dis..

[9] Hevroni G., Skwiersky S., Zhyvotovska A., McFarlane S.I. (2020). Metformin Use and the Risk of Gastrointestinal Malignancies in Diabetic Populations: A Meta-Analysis. Int. J. Clin. Endocrinol. Metab..

[10] Umezawa S., Higurashi T., Komiya Y., Arimoto J., Horita N., Kaneko T., Iwasaki M., Nakagama H., Nakajima A. (2019). Chemoprevention of Colorectal Cancer: Past, Present, and Future. Cancer Sci..

[11] Ala M. (2022). The Emerging Role of Metformin in the Prevention and Treatment of Colorectal Cancer: A Game Changer for the Management of Colorectal Cancer. Curr. Diabetes Rev..

[12] Deng M., Lei S., Huang D., Wang H., Xia S., Xu E., Wu Y., Zhang H. (2020). Suppressive Effects of Metformin on Colorectal Adenoma Incidence and Malignant Progression. Pathol. Res. Pract..

[13] Lee J.-W., Choi E.-A., Kim Y.-S., Kim Y., You H.-S., Han Y.-E., Kim H.-S., Bae Y.-J., Kim J., Kang H.-T. (2021). Metformin Usage and the Risk of Colorectal Cancer: A National Cohort Study. Int. J. Color. Dis..

[14] Jeong G.H., Lee K.H., Kim J.Y., Eisenhut M., Kronbichler A., van der Vliet H.J., Hong S.H., Shin J.I., Gamerith G. (2019). Effect of Statin on Cancer Incidence: An Umbrella Systematic Review and Meta-Analysis. J. Clin. Med..

[15] Lytras T., Nikolopoulos G., Bonovas S. (2014). Statins and the Risk of Colorectal Cancer: An Updated Systematic Review and Meta-Analysis of 40 Studies. World J. Gastroenterol..

[16] Ibáñez-Sanz G., Guinó E., Pontes C., Quijada-Manuitt M.Á., de la Peña-Negro L.C., Aragón M., Domínguez M., Rodríguez-Alonso L., Blasco A., García-Rodríguez A. (2019). Statin Use and the Risk of Colorectal Cancer in a Population-Based Electronic Health Records Study. Sci. Rep..

[17] Ibáñez-Sanz G., Guinó E., Pontes C., Morros R., de la Peña-Negro L.C., Quijada-Manuitt M.Á., Moreno V. (2020). Risk of Colorectal Cancer in Users of Bisphosphonates: Analysis of Population-Based Electronic Health Records. Eur. J. Epidemiol..

[18] Thosani N., Thosani S.N., Kumar S., Nugent Z., Jimenez C., Singh H., Guha S. (2013). Reduced Risk of Colorectal Cancer with Use of Oral Bisphosphonates: A Systematic Review and Meta-Analysis. J. Clin. Oncol. Off. J. Am. Soc. Clin. Oncol..

[19] Aran D., Camarda R., Odegaard J., Paik H., Oskotsky B., Krings G., Goga A., Sirota M., Butte A.J. (2017). Comprehensive Analysis of Normal Adjacent to Tumor Transcriptomes. Nat. Commun..

[20] Sanz-Pamplona R., Berenguer A., Cordero D., Molleví D.G., Crous-Bou M., Sole X., Paré-Brunet L., Guino E., Salazar R., Santos C. (2014). Aberrant Gene Expression in Mucosa Adjacent to Tumor Reveals a Molecular Crosstalk in Colon Cancer. Mol. Cancer.

[21] Dampier C.H., Devall M., Jennelle L.T., Díez-Obrero V., Plummer S.J., Moreno V., Casey G. (2020). Oncogenic Features in Histologically Normal Mucosa: Novel Insights Into Field Effect From a Mega-Analysis of Colorectal Transcriptomes. Clin. Transl. Gastroenterol..

[22] Bien S.A., Su Y.-R., Conti D.V., Harrison T.A., Qu C., Guo X., Lu Y., Albanes D., Auer P.L., Banbury B.L. (2019). Genetic Variant Predictors of Gene Expression Provide New Insight into Risk of Colorectal Cancer. Hum. Genet..

[23] Vertzoni M., Augustijns P., Grimm M., Koziolek M., Lemmens G., Parrott N., Pentafragka C., Reppas C., Rubbens J., Van Den Abeele J. (2019). Impact of Regional Differences along the Gastrointestinal Tract of Healthy Adults on Oral Drug Absorption: An UNGAP Review. Eur. J. Pharm. Sci..

[24] Burgess S., Thompson S.G. (2013). Use of Allele Scores as Instrumental Variables for Mendelian Randomization. Int. J. Epidemiol..

[25] Davey Smith G., Hemani G. (2014). Mendelian Randomization: Genetic Anchors for Causal Inference in Epidemiological Studies. Hum. Mol. Genet..

[26] Walker V.M., Davey Smith G., Davies N.M., Martin R.M. (2017). Mendelian Randomization: A Novel Approach for the Prediction of Adverse Drug Events and Drug Repurposing Opportunities. Int. J. Epidemiol..

[27] Yarmolinsky J., Díez-Obrero V., Richardson T.G., Pigeyre M., Sjaarda J., Paré G., Walker V.M., Vincent E.E., Tan V.Y., Obón-Santacana M. (2022). Genetically Proxied Therapeutic Inhibition of Antihypertensive Drug Targets and Risk of Common Cancers: A Mendelian Randomization Analysis. PLoS Med..

[28] Díez-Obrero V., Dampier C.H., Moratalla-Navarro F., Devall M., Plummer S.J., Díez-Villanueva A., Peters U., Bien S., Huyghe J.R., Kundaje A. (2021). Genetic Effects on Transcriptome Profiles in Colon Epithelium Provide Functional Insights for Genetic Risk Loci. Cell. Mol. Gastroenterol. Hepatol..

[29] Díez-Obrero V., Moratalla-Navarro F., Dampier C., Devall M., Carreras-Torres R., Casey G., Moreno V. (2021). The Colon Transcriptome Explorer (CoTrEx) 2.0: A Reference Web-Based Resource for Exploring Population-Based Normal Colon Gene Expression. Preprints.

[30] Momozawa Y., Dmitrieva J., Théâtre E., Deffontaine V., Rahmouni S., Charloteaux B., Crins F., Docampo E., Elansary M., Gori A.-S. (2018). IBD Risk Loci Are Enriched in Multigenic Regulatory Modules Encompassing Putative Causative Genes. Nat. Commun..

[31] The Gtex Consortium (2020). The GTEx Consortium Atlas of Genetic Regulatory Effects across Human Tissues. Science.

[32] Burgess S., Dudbridge F., Thompson S.G. (2016). Combining Information on Multiple Instrumental Variables in Mendelian Randomization: Comparison of Allele Score and Summarized Data Methods. Stat. Med..

[33] Subramanian A., Tamayo P., Mootha V.K., Mukherjee S., Ebert B.L., Gillette M.A., Paulovich A., Pomeroy S.L., Golub T.R., Lander E.S. (2005). Gene Set Enrichment Analysis: A Knowledge-Based Approach for Interpreting Genome-Wide Expression Profiles. Proc. Natl. Acad. Sci. USA.

[34] Pickup M.W., Mouw J.K., Weaver V.M. (2014). The Extracellular Matrix Modulates the Hallmarks of Cancer. EMBO Rep..

[35] Popova N.V., Jücker M. (2022). The Functional Role of Extracellular Matrix Proteins in Cancer. Cancers.

[36] Winkler J., Abisoye-Ogunniyan A., Metcalf K.J., Werb Z. (2020). Concepts of Extracellular Matrix Remodelling in Tumour Progression and Metastasis. Nat. Commun..

[37] LaMoia T.E., Shulman G.I. (2021). Cellular and Molecular Mechanisms of Metformin Action. Endocr. Rev..

[38] Riedesel M.L., Williams B.A. (1976). Continuous 24-Hour Oxygen Consumption Studies of Myotis Velifer. Comp. Biochem. Physiol. A.

[39] Chen X., Yi C.-H., Ya K.-G. (2020). Renin-Angiotensin System Inhibitor Use and Colorectal Cancer Risk and Mortality: A Dose-Response Meta Analysis. J. Renin-Angiotensin-Aldosterone Syst. JRAAS.

[40] Qi J., An R., Bhatti P., Spinelli J.J., Murphy R.A. (2022). Anti-Hypertensive Medications and Risk of Colorectal Cancer: A Systematic Review and Meta-Analysis. Cancer Causes Control CCC.

[41] Zhang Y., Song M., Chan A.T., Meyerhardt J.A., Willett W.C., Giovannucci E.L. (2022). Long-Term Use of Antihypertensive Medications, Hypertension and Colorectal Cancer Risk and Mortality: A Prospective Cohort Study. Br. J. Cancer.

[42] Okwan-Duodu D., Landry J., Shen X.Z., Diaz R. (2013). Angiotensin-Converting Enzyme and the Tumor Microenvironment: Mechanisms beyond Angiogenesis. Am. J. Physiol. Regul. Integr. Comp. Physiol..

[43] Kim H., Hur J., Wu K., Song M., Wang M., Smith-Warner S.A., Zhang X., Giovannucci E.L. (2023). Total Calcium, Dairy Foods and Risk of Colorectal Cancer: A Prospective Cohort Study of Younger US Women. Int. J. Epidemiol..

[44] Lopez-Caleya J.F., Ortega-Valín L., Fernández-Villa T., Delgado-Rodríguez M., Martín-Sánchez V., Molina A.J. (2022). The Role of Calcium and Vitamin D Dietary Intake on Risk of Colorectal Cancer: Systematic Review and Meta-Analysis of Case-Control Studies. Cancer Causes Control CCC.

[45] Karavasiloglou N., Hughes D.J., Murphy N., Schomburg L., Sun Q., Seher V., Rohrmann S., Weiderpass E., Tjønneland A., Olsen A. (2023). Prediagnostic Serum Calcium Concentrations and Risk of Colorectal Cancer Development in 2 Large European Prospective Cohorts. Am. J. Clin. Nutr..

[46] Devall M.A.M., Dampier C.H., Eaton S., Ali M.W., Plummer S.J., Bryant J., Gauderman W.J., Peters U., Powell S.M., Casey G. (2022). Transcriptomic Response to Calcium in Normal Colon Organoids Is Impacted by Colon Location and Sex. Cancer Prev. Res..

[47] Wu J.-Y., Shao Y., Huang C.-Z., Wang Z.-L., Zhang H.-Q., Fu Z. (2023). Genetic Variants in the Calcium Signaling Pathway Participate in the Pathogenesis of Colorectal Cancer through the Tumor Microenvironment. Front. Oncol..

[48] Iqbal U., Nguyen P.-A., Syed-Abdul S., Yang H.-C., Huang C.-W., Jian W.-S., Hsu M.-H., Yen Y., Li Y.-C. (2015). (Jack). Is Long-Term Use of Benzodiazepine a Risk for Cancer?. Medicine.

[49] Kim H.-B., Myung S.-K., Park Y.C., Park B. (2017). Use of Benzodiazepine and Risk of Cancer: A Meta-Analysis of Observational Studies. Int. J. Cancer.

[50] Zhang T., Yang X., Zhou J., Liu P., Wang H., Li A., Zhou Y. (2017). Benzodiazepine Drug Use and Cancer Risk: A Dose-Response Meta Analysis of Prospective Cohort Studies. Oncotarget.

[51] Cui Y., Wen W., Zheng T., Li H., Gao Y.-T., Cai H., You M., Gao J., Yang G., Zheng W. (2019). Use of Antihypertensive Medications and Survival Rates for Breast, Colorectal, Lung, or Stomach Cancer. Am. J. Epidemiol..

[52] Li L., Cui N., Hao T., Zou J., Jiao W., Yi K., Yu W. (2021). Statins Use and the Prognosis of Colorectal Cancer: A Meta-Analysis. Clin. Res. Hepatol. Gastroenterol..

[53] Barclay N., Tarallo M., Hendrikx T., Marett S. (2013). Patient Preference for Oral Versus Injectable and Intravenous Methods of Treatment for Rheumatoid Arthritis. Value Health.

[54] Eek D., Krohe M., Mazar I., Horsfield A., Pompilus F., Friebe R., Shields A.L. (2016). Patient-Reported Preferences for Oral versus Intravenous Administration for the Treatment of Cancer: A Review of the Literature. Patient Prefer. Adherence.

[55] Cui G., Wang Z., Liu H., Pang Z. (2022). Cytokine-Mediated Crosstalk between Cancer Stem Cells and Their Inflammatory Niche from the Colorectal Precancerous Adenoma Stage to the Cancerous Stage: Mechanisms and Clinical Implications. Front. Immunol..

[56] Pellatt A.J., Slattery M.L., Mullany L.E., Wolff R.K., Pellatt D.F. (2016). Dietary Intake Alters Gene Expression in Colon Tissue: Possible Underlying Mechanism for the Influence of Diet on Disease. Pharmacogenet. Genom..

[57] Slattery M.L., Pellatt D.F., Mullany L.E., Wolff R.K. (2015). Differential Gene Expression in Colon Tissue Associated With Diet, Lifestyle, and Related Oxidative Stress. PLoS ONE.

[58] Brumpton B., Sanderson E., Heilbron K., Hartwig F.P., Harrison S., Vie G.Å., Cho Y., Howe L.D., Hughes A., Boomsma D.I. (2020). Avoiding Dynastic, Assortative Mating, and Population Stratification Biases in Mendelian Randomization through within-Family Analyses. Nat. Commun..

[59] Obón-Santacana M., Moratalla-Navarro F., Guinó E., Carreras-Torres R., Díez-Obrero V., Bars-Cortina D., Ibáñez-Sanz G., Rodríguez-Alonso L., Mata A., García-Rodríguez A. (2024). Diet Impacts on Gene Expression in Healthy Colon Tissue: Insights from the BarcUVa-Seq Study. Nutrients.

[60] World Health Organization The ICD-10 Classification of Mental and Behavioural Disorders: Diagnostic Criteria for Research. https://www.who.int/classifications/classification-of-diseases.

[61] World Health Organization Anatomical Therapeutic Chemical (ATC) Classification System. http://www.whocc.no/atc/.

[62] Robinson M.D., Oshlack A. (2010). A Scaling Normalization Method for Differential Expression Analysis of RNA-Seq Data. Genome Biol..

[63] Díez-Obrero V., Moratalla-Navarro F., Ibáñez-Sanz G., Guardiola J., Rodríguez-Moranta F., Obón-Santacana M., Díez-Villanueva A., Dampier C.H., Devall M., Carreras-Torres R. (2022). Transcriptome-Wide Association Study for Inflammatory Bowel Disease Reveals Novel Candidate Susceptibility Genes in Specific Colon Subsites and Tissue Categories. J. Crohns Colitis.

[64] Ritchie M.E., Phipson B., Wu D., Hu Y., Law C.W., Shi W., Smyth G.K. (2015). Limma Powers Differential Expression Analyses for RNA-Sequencing and Microarray Studies. Nucleic Acids Res..

[65] Yu G., He Q.-Y. (2016). ReactomePA: An R/Bioconductor Package for Reactome Pathway Analysis and Visualization. Mol. Biosyst..

[66] Robinson M.D., McCarthy D.J., Smyth G.K. (2010). edgeR: A Bioconductor Package for Differential Expression Analysis of Digital Gene Expression Data. Bioinformatics.

[67] Wishart D.S., Knox C., Guo A.C., Shrivastava S., Hassanali M., Stothard P., Chang Z., Woolsey J. (2006). DrugBank: A Comprehensive Resource for in Silico Drug Discovery and Exploration. Nucleic Acids Res..

[68] Yengo L., Sidorenko J., Kemper K.E., Zheng Z., Wood A.R., Weedon M.N., Frayling T.M., Hirschhorn J., Yang J., Visscher P.M. (2018). Meta-Analysis of Genome-Wide Association Studies for Height and Body Mass Index in ~700,000 Individuals of European Ancestry. Hum. Mol. Genet..

[69] Liu M., Jiang Y., Wedow R., Li Y., Brazel D.M., Chen F., Datta G., Davila-Velderrain J., McGuire D., Tian C. (2019). Association Studies of up to 1.2 Million Individuals Yield New Insights into the Genetic Etiology of Tobacco and Alcohol Use. Nat. Genet..

[70] Doherty A., Smith-Byrne K., Ferreira T., Holmes M.V., Holmes C., Pulit S.L., Lindgren C.M. (2018). GWAS Identifies 14 Loci for Device-Measured Physical Activity and Sleep Duration. Nat. Commun..

