# Supplementary figures and images for: Exploring the Chemopreventive Effect of Medication on Gene Expression Linked to Colorectal Cancer: An Observational and Mendelian Randomization Analysis in Healthy Colon Mucosa
Source: Int J Mol Sci. 2024 Oct 23;25(21):11395. doi: 10.3390/ijms252111395 (PMC11547083; doi:10.3390/ijms252111395)

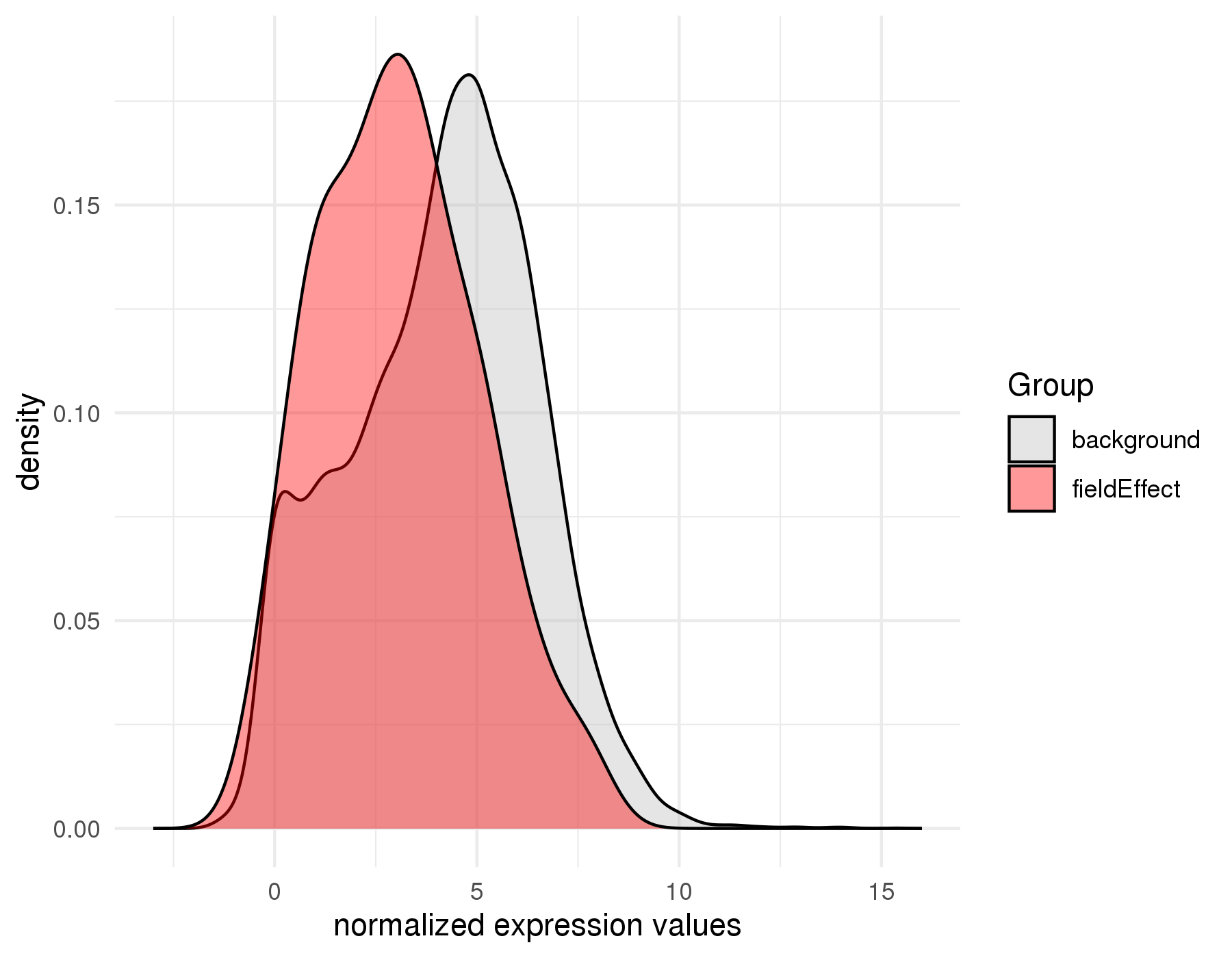

Supplement: Supplementary file 1 [file ijms-25-11395-s001.zip › supplementaryFiles/Supplemental Figure S1.png]

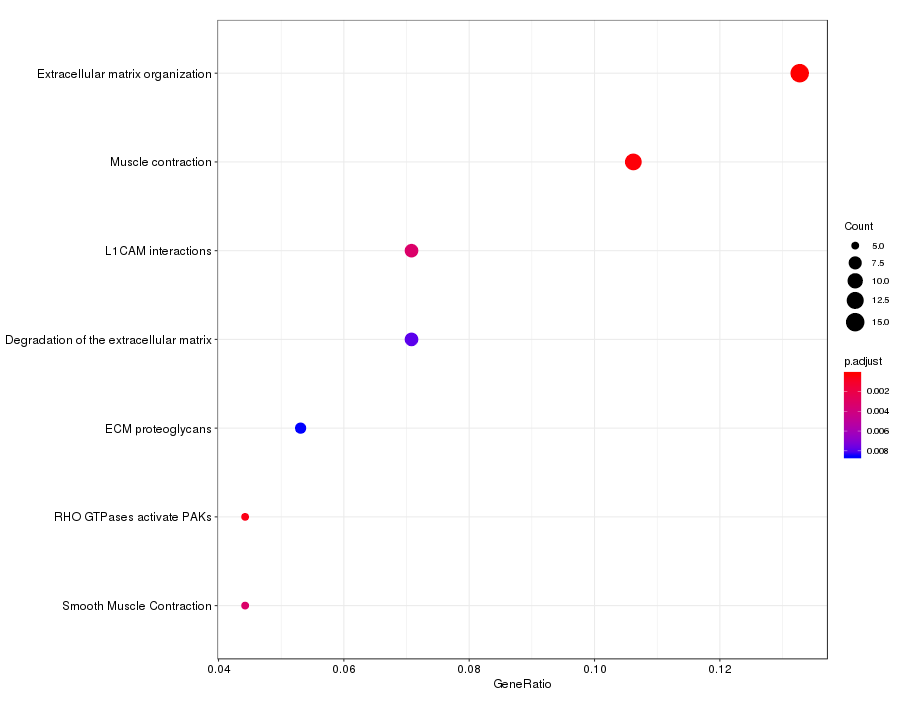

Supplement: Supplementary file 1 [file ijms-25-11395-s001.zip › supplementaryFiles/Supplemental Figure S2.png]

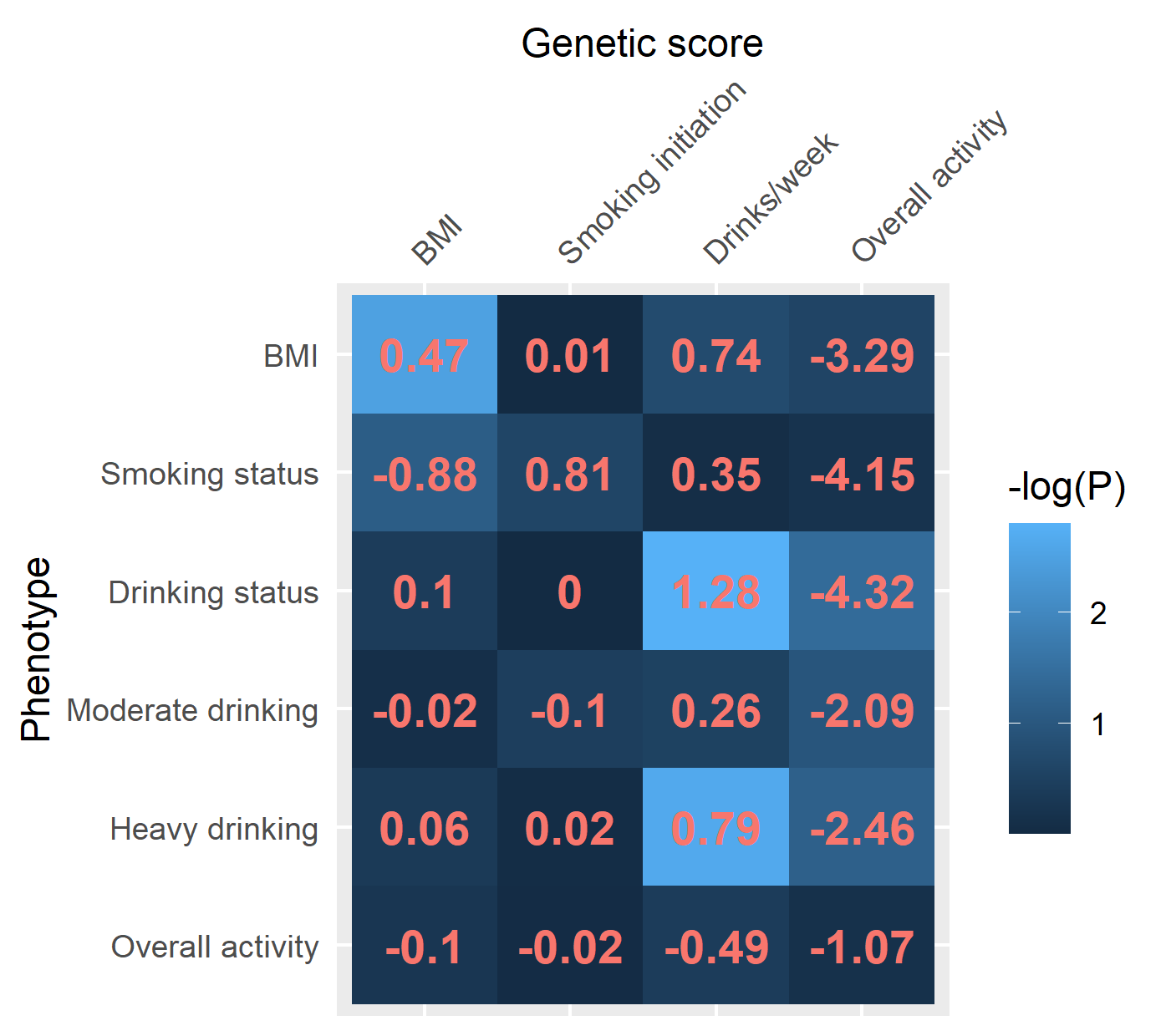

Supplement: Supplementary file 1 [file ijms-25-11395-s001.zip › supplementaryFiles/Supplemental Figure S3.tiff]
